# Ultrasonographic diagnosis of adnexal masses: interobserver agreement in the interpretation of videos, using IOTA terminology

**DOI:** 10.1007/s00404-023-07233-z

**Published:** 2023-10-04

**Authors:** Roberta Massobrio, Luca Liban Mariani, Daniele Conti, Tiziana De Grandis, Francesca Buonomo, Enrico Badellino, Lorenzo Novara, Valentina Elisabetta Bounous, Stefania Perotto, Matteo Mancarella, Annamaria Ferrero, Nicoletta Biglia, Luca Fuso

**Affiliations:** 1 Academic Division of Gynecology and Obstetrics, Mauriziano Hospital, University of Torino, 10128 Turin, Italy; 2 SynDiag srl, c/o Innovative Enterprises Incubator of Polytechnic University of Turin, 10129 Turin, Italy; 3 Candiolo Cancer Institute, FPO-IRCCS-Candiolo, 10060 Turin, Italy; 4 Institute for Maternal and Child Health - IRCCS ‘‘Burlo Garofolo’’, 34137 Trieste, Italy; 5 Division of Gynecologic Oncology, Michele e Pietro Ferrero Hospital, 12060 Verduno, Italy

**Keywords:** Gynecological ultrasound, Ovarian tumors, IOTA models, IOTA terminology

## Abstract

**Objectives:**

Aim of this study is to estimate interobserver agreement in classifying adnexal tumors using IOTA terms, simple rules and subjective assessment. In addition, we related observers’ accuracy with their experience in gynecological ultrasonography and the year of IOTA certification.

**Methods:**

Eleven observers with three different levels of experience evaluated videoclips of 70 adnexal masses, defining tumor type according to IOTA terms and definitions, classifying the mass using IOTA Simple rules and Subjective assessment as well as providing Color Score evaluation. Sensitivity, specificity and area under the ROC curve were calculated and the year of IOTA certification was related with operators’ accuracy through Pearson correlation coefficient. Interobserver agreement was estimated calculating percentage of agreement, Fleiss kappa and Cohen’s kappa.

**Results:**

We found a positive correlation between the year of IOTA certification and operators’ accuracy (Pearson coefficient 0.694), especially among the observers with the least experience, the residents (*p* = 0.003). For tumor type classification, identification of papillary projections and classification of tumors using subjective assessment, agreement among all observers was moderate (Fleiss kappa 0.455, 0.552, and 0.476, respectively) and increased with the years of experience. Agreement in the application of Simple Rules was moderate in all examiners with IOTA certification, with Fleiss kappa in the range of (0.403, 0.498). For Color Score assignment interobserver agreement among all observers was fair (Cohen’s kappa 0.380).

**Conclusions:**

Even among expert examiners, the results of adnexal lesion assessment can be inconsistent. Experience impacts on accuracy and agreement in subjective assessment, while the application of Simple Rules can mitigate the role of experience in interobserver agreement. The knowledge of IOTA models among residents seams to improve their diagnostic accuracy, showing the benefits of IOTA terminology for in training sonographers.

## What does this study add to the clinical work

**Table:** 

The application of IOTA Simple Rules can mitigate the role of experience in interobserver agreement in adnexal tumors ultrasonographic assessment. IOTA models have an implication for training in less experienced examiners, improving their diagnostic accuracy.	

## Introduction

Distinguishing between benign and malignant adnexal masses can be challenging for sonographers. This differentiation is crucial to determine whether conservative management or surgical treatment is necessary, as well as to determine what level of expertise is required for the surgery.

To ensure an accurate evaluation of adnexal lesions, it is essential to adhere to a standardized procedure [1] that focuses on identifying the most crucial features for accurate differential diagnosis. This evaluation process is aided by both the operator's ultrasound examination experience and the implementation of IOTA (International Ovarian Tumor Analysis) terminology and models. These tools provide guidance to both expert operators and those with less experience, effectively reducing the gap in their ability to define the risk of malignancy.

According to IOTA terminology [2], lesions can be classified as unilocular, unilocular-solid, multilocular, multilocular-solid or solid, depending on the presence of septa and solid components. Accurate classification of each mass is dependent on the identification of papillary projections, which can significantly impact the assessment of malignancy risk. Therefore, it is crucial to identify these projections during the evaluation process [3]. While Color Doppler examination may have limited value in the differential diagnosis of certain conditions, it can still play a role in increasing the confidence with which a correct diagnosis is made [4]. The IOTA Color Score is a useful tool for expressing vascular features of adnexal lesions. By assigning a score ranging from 1 (indicating no vascularization) to 4 (indicating abundant vascularization), it allows for the semi-quantitative description of the amount of blood flow within the lesion [2].

IOTA ADNEX model [5] and Simple Rules [6] have been validated as useful tools to guide clinicians in the examination of adnexal lesions. While some studies have suggested that subjective assessment may be superior to mathematical models in the classification of these tumors [7], the IOTA models have shown comparable performance to expert evaluation [8]. Furthermore, these models can be utilized by sonographers of varying levels of experience, making them a valuable resource in the diagnostic process.

Despite the introduction of IOTA models in clinical practice, interpreting adnexal masses during ultrasound examination remains a challenge. This difficulty has resulted in variability among sonographers in identifying features that are associated with benign or malignant tumors, leading to non-uniform definitions of the tumors being analyzed [9].

The aim of our study is to estimate interobserver agreement between examiners with different levels of experience in classifying adnexal tumors using IOTA terminology and subjective assessment, in assessing the presence of papillary projections and the quantification of Color Score. In addition, we looked for a correlation of observers’ accuracy and their experience in gynecological ultrasonography and the year of IOTA certification.

## Methods

This is a prospective multicentric observational study. Eleven sonographers participated, in the role of observers: group 1 had four observers (A, B, C and D) who were gynecologists with more than 10 year experience in gynecological ultrasonography and a special interest in adnexal masses, working in three different level II centers (IRCCS of Candiolo, Gynecology and Obstetrics Department in Mauriziano Hospital, Torino and IRCCS Burlo Garofolo, Trieste); group 2 had three observers (E, F, G), two from the Gynecology and Obstetrics Department in Mauriziano Hospital, Turin and one from the IRCCS of Candiolo, who had between three and ten years of experience in transvaginal ultrasound; group 3 had four observers who were residents in Obstetrics and Gynecology (observers H, I, L, M), two from the Gynecology and Obstetrics Department in Mauriziano Hospital, Torino and two from the IRCCS Burlo Garofolo, Trieste, who have been performing transvaginal ultrasonography for less than 3 years. In group 3, two observers had IOTA certification, while two were not certified by IOTA.

Video clips of 70 adnexal masses from 68 patients were consecutively acquired by two sonographers with more than 10 year experience trained in IOTA terms and techniques, one of the Gynecology and Obstetrics Department in Mauriziano Hospital, Torino, and one of the IRCCS of Candiolo during their clinical practice.

All patients underwent surgical treatment and a histopathological diagnosis was available for all the lesions included.

The sonographers who generated the videos did not participate otherwise in the evaluation of the masses. Affiniti 50 or Affiniti 70 ultrasound machines (Philips, Amsterdam, The Netherlands, 2013) equipped either with a C9 − 4 v Endocavitary Probe with a 4.0–9.0 MHz frequency range or with a C10 − 3v Endocavitary Probe with a 3.0–10.0 MHz frequency range were used. For all the adnexal tumors included, diagnosis had been confirmed by anatomopathological examination.

After the collection phase, all videos were reviewed, to ensure that the entire mass was clearly visualized, and that the duration of the clip was between 5 and 10 s. For 12 out of the 70 adnexal lesions included, Color/Power Doppler videos were not considered adequate for the evaluation, therefore only a grayscale video was provided. For most of the masses (58 out of 70) two videos were included: one in grayscale and the second video in Color/Power Doppler mode. Additional information provided to observers during the evaluation of adnexal lesions included the maximum diameter of the lesion, a description and size of any solid components present, the patient's menopausal status and the Color Score. The latter was provided only in cases where a second clip in Color/Power Doppler mode was not available. The maximum diameter and the Color Score indicated were defined by the sonographer who performed the examination at the moment of the videoclip acquisition.

All the observers were asked to evaluate each adnexal mass using IOTA terminology and models. In addition, they were asked to disclose their years of experience in gynecological ultrasound and, for those that had been IOTA certified, the year of their IOTA certification. Educational material on IOTA terminology and models was presented before starting the evaluation [10].

The observers were instructed to evaluate each adnexal mass and classify it according to IOTA terminology and definitions (unilocular, unilocular solid, multilocular, multilocular solid, solid). They were also asked to assess the presence of papillary projections and, if possible, apply the Simple Rules. In addition, classification of the masses as benign or malignant using subjective assessment and Color Score assignment (if an image in Doppler mode had been provided) were required. In the evaluation of lesions according to subjective assessment, borderline tumors were classified as malignant. Each observer was unaware of the responses of the others and of the histopathological diagnosis of the masses under examination.

The study was conducted in accordance with the Declaration of Helsinki and approved by the Institutional Review Board and Ethics Committee A.O.U. Città della Salute e della Scienza di Torino/A.O. Ordine Mauriziano/ASL Città di Torino (registration number 58563).

### Statistical analysis

Interobserver agreement was estimated calculating the percentage of agreement for each observers pair and then averaging the results (mean percentage agreement), calculating Fleiss kappa [11] for multiple observers and Cohen’s kappa [12] for each observers pair, with mean and range of Cohen’s kappa values reported. Results are presented for all observers as a whole group and separately for the three groups of experience described above. Kappa values between 0.81 and 1 indicate very good agreement, between 0.61 and 0.80 good agreement, between 0.41 and 0.60 moderate, between 0.21 and 0.40 of fair agreement and < 0.20 poor agreement [13], according to Landis and Koch guidelines [14]. To test the differences between Fleiss k values according observers’ experience, a *Z* test statistic was performed, with 2-sided p values. *P* value < 0.05 was considered significant. For interobserver agreement with regard to Color Score assignment Fleiss Kappa was not calculated, since only for 58 lesions a second clip in color/power Doppler mode was provided.

The diagnostic accuracy of each observer was analyzed, relating the classification of the mass as benign or malignant through Subjective Assessment and the histopathological diagnosis, which was considered as the gold standard. Sensitivity, specificity, positive predictive value (PPV), negative predictive value (NPV), accuracy and area under the ROC curve were used as accuracy indicators. In addition, the impact of experience in application of IOTA terminology on operators’ accuracy was assessed, calculating the Pearson correlation coefficient between the two variables.

The analysis was performed using statistical package SPSS Ver. 17 for Windows (Chicago, IL); Cohen’s kappa and Fleiss kappa values were obtained through the additional component for Microsoft Excel "Real Statistics".

## Results

Video clips of 70 adnexal masses from 68 patients (2 with bilateral masses) were included in the study. The average age of patients was 51 years (range 18–80) and 33 (48.5%) were postmenopausal. Table 1 shows the histological diagnosis of the 70 masses included: 46 (65.7%) were benign and 24 (34.3%) malignant, in particular 13 (18.6%) borderline, 10 (14.3%) invasive and 1 (1.4%) was an ovarian metastasis from intestinal mucinous adenocarcinoma.

**Table 1:** Histological diagnosis of the 70 masses included

Diagnosis	*n*
Benign masses	
Endometrioma	7
Teratoma	4
Serous cystadenoma	10
Mucinous cystadenoma	6
Sero-mucinous cystadenoma	2
Cystadenofibroma	5
Fibrothecoma	2
Ovarian fibroma	2
Luteal cyst	2
Simple cyst	2
Paraovarian serous cyst	4
Borderline masses	
Serous papillary borderline tumor	9
Mucinous borderline tumor	3
Borderline mucinous cystadenofibroma	1
Primary invasive masses	
Papillary serous cystadenocarcinoma	5
Mucinous cystadenocarcinoma	1
Endometrioid adenocarcinoma	1
Clear-cell carcinoma	1
Undifferentiated carcinoma	1
Carcinosarcoma	1
Ovarian metastases	
Intestinal mucinous adenocarcinoma	1

Interobserver agreement on tumor type classification and the presence of papillary projections, estimated by percentage of agreement, Fleiss Kappa and Cohen’s kappa values is reported in Table 2.

**Table 2:** Interobserver agreement with regard to tumor type (unilocular, unilocular solid, multilocular solid, solid) and presence of papillary projection. Group 1: experienced observers, group 2: moderately experienced observers, group 3: residents

Parameter	Agreement (%)	Fleiss kappa	Cohen’s kappa	Agreement (%)	Fleiss kappa	Cohen’s kappa
	Tumor type			Papillary projections		
All observers (*n* = 11)	57.7 (34.3–80.0)	0.455 (0.438–0.471)	0.454 (0.135–0.745)	81.5% (68.6–90.0)	0.552 (0.521–0.584)	0.555 (0.295–0.787)
Group 1 (*n* = 4)	68.1 (60.0–80.0)	0.592 (0.543–0.641)	0.594 (0.495–0.745)	86.67 (82.9–90.0)	0.694 (0.599–0.790)	0.696 (0.606–0.787)
Group 2 (*n* = 3)	59.5 (58.6–61.4)	0.482 (0.412–0.552)	0.485 (0.467–0.510)	80.0 (78.6–81.4)	0.510 (0.375–0.645)	0.511 (0.496–0.541)
Group 3 (*n* = 4)	51.4 (35.7–72.9)	0.358 (0.306–0.410)	0.362 (0.166–0.654)	79.7 (68.6–87.1)	0.484 (0.388–0.580)	0.503 (0.295–0.630)

Interobserver agreement for classifying tumor types among all of observers was 57.7%. The Fleiss kappa statistic yielded a value of 0.455, which represents a moderate agreement. Further analysis within specific groups revealed interesting patterns. In group 1, there was a moderate level of agreement with a Fleiss kappa coefficient of 0.592. However, in group 2 agreement significantly decreased (Fleiss kappa 0.482, *p* = 0.005), indicating less consensus compared to group 1. Similarly, in the residents group, agreement was fair with a Fleiss kappa coefficient of 0.358 (*p* = 0.0001), lower than that observed in both group 1 and group 2.

The interobserver agreement among the eleven observers with regard to detection of papillary projections was determined to be moderate, with an agreement of 81.5% and a Fleiss kappa coefficient of 0.522. In group 1, the agreement among observers was good, with a Fleiss kappa of 0.694. However, as for the less experienced groups, the agreement decreased: group 2 showed a moderate agreement (Fleiss kappa 0.510, *p* = 0.01), and group 3 demonstrated a slightly lower agreement (Fleiss kappa 0.484, *p* = 0.001).

The interobserver agreement for the classification of masses according to Simple Rules as benign, malignant, or inconclusive was found to be moderate among all observers, with a Fleiss kappa coefficient of 0.491 (Table 3). Upon subgroup analysis, there was a moderate agreement among observers in group 1 (Fleiss kappa 0.498), similar to the agreement observed among observers in group 2 (Fleiss kappa 0.498, *p* = 0.37). However, within group 3 there were varying agreement levels between the observers with IOTA certification (H and I), who demonstrated a moderate agreement (Cohen's kappa 0.438), and the remaining two residents, who showed a fair agreement (Cohen's kappa 0.318).

**Table 3:** Interobserver agreement with regard to the classification of lesions according to IOTA Simple Rules and subjective assessment. Group 1: experienced observers, group 2: moderately experienced observers, group 3: residents; a: residents certified by IOTA, b: residents not certified by IOTA

Parameter	Agreement (%)	Fleiss kappa	Cohen’s kappa	Agreement (%)	Fleiss kappa	Cohen’s kappa
	Simple rules			Subjective assessment		
All observers (*n* = 11)	70.0 (57.1–81.4)	0.491 (0.468–0.514)	0.490 (0.275–0.689)	68.2 (41.4–87.1)	0.476 (0.453–0.499)	0.492 (0.206–0.763)
Group 1 (*n* = 4)	71.0 (65.7–78.6)	0.498 (0.428–0.569)	0.499 (0.399–0.624)	84.0 (80.0–87.1)	0.702 (0.629–0.775)	0.702 (0.626–0.763)
Group 2 (*n* = 3)	70.9 (64.3–74.3)	0.518 (0.415–0.615)	0.518 (0.412–0.573)	81.4 (77.1–85.7)	0.676 (0.575–0.778)	0.677 (0.602–0.745)
Group 3 (*n* = 4)	65.5 (57.1–74.3)	0.400 (0.330–0.571)	0.403 (0.275–0.570) (a) 0.438 (b) 0.317	56.9 (44.3–72.8)	0.326 (0.226–0.395)	0.347 (0.206–0.563) (a) 0.410 (b) 0.563

The interobserver agreement among all observers for the subjective assessment of tumors as benign or malignant was determined to be moderate, with a percentage agreement of 68.2% and a Fleiss kappa coefficient of 0.476. It is important to note that this level of agreement is lower than the agreement observed in the classification of masses according to Simple Rules. In specific groups, both group 1 and group 2 demonstrated better agreement in the subjective assessment of tumors (Fleiss kappa 0.702 and 0.676, respectively) compared to their agreement in the classification through the application of Simple Rules. Although the discrepancy in agreement between observers in groups 1 and 2 was minimal (*p* = 0.034), it increased notably in group 3 (Fleiss kappa 0.347, *p* = 0.00001). This indicates a decreased level of agreement in the subjective assessment of tumors within group 3 compared to the other two groups.

Table 4 presents the interobserver agreement in assigning a Color Score (1, 2, 3, or 4) for tumors using a video that included the Color/Power Doppler mode. The agreement among all observers was determined to be fair, with a mean Cohen's kappa value of 0.380 calculated for all possible pairs of observers. Among the expert sonographers in group 1, the concordance was higher, with an average agreement of 60.9% and a Cohen's kappa value of 0.460, indicating a moderate level of concordance between expert operators. However, both the percentage of agreement and Cohen's kappa were lower for group 2 (mean Cohen's kappa 0.374, *p* = 0.037) and group 3 (mean Cohen's kappa 0.348, *p* = 0.001), indicating a fair level of agreement within these groups.

**Table 4:** Interobserver agreement IOTA color score assessment group 1: experienced observers, group 2: moderately experienced observers, group 3: residents

Parameter	Agreement (%)	Cohen’s kappa
All observers (*n* = 11)	54.5 (42.3–67.3)	0.380 (0.226–0.542)
Group 1 (*n* = 4)	60.9 (57.1–64.6)	0.460 (0.399–0.514)
Group 2 (*n* = 3)	53.3 (46.1–62.7)	0.374 (0.260–0.508)
Group 3 (*n* = 4)	52.0 (44.2–56.0)	0.348 (0.266–0.403)

Sensitivity, specificity, VPP, VPN, accuracy and area under the ROC curve (AUC) of each observer were calculated (Table 5). The relationship between observers’ accuracy and number of months since IOTA certification was evaluated through a linear regression. We noticed that observers with more experience with IOTA terminology and models were more accurate, with a Pearson coefficient of 0.694 (*p* = 0.018). The linear correlation was more evident among the less expert observers: the difference in terms of AUC between the two residents using IOTA models and the two residents without IOTA certification was statistically significant (*p* = 0.003). Specifically, observers in group 1, the most experienced ones, showed sensitivity, specificity and values of AUC in the range of (88%, 96%), (91%, 93%) and (0.907, 0.947), respectively. Observers in group 2 showed sensitivity, specificity and values of AUC in the range of (72%, 96%), (80%, 82%) and (0.760, 0.891), respectively. Finally, observers in group 3 showed sensitivity, specificity and values of AUC in the range of (100%, 88%), (42%, 87%) and (0.691, 0.889) with a substantial difference among observers with IOTA certification and those without IOTA certification. The former showed specificity and AUC with a range of 78–87% and 0.760–0.889, respectively, with a confidence interval for specificity being 68–93%, while the latter showed specificity and AUC with a range of 42–44% and 0.691–0.702, respectively, with the confidence interval for specificity being 28–59%.

**Table 5:** Observers’ accuracy, years of experience, year of IOTA certification. Observers A, B, C, D belong to group 1; observers E, F, G belong to group 2; observers H, I, L, M belong to group 3

Observer	Sensitivity %	Specificity %	NPV%	PPV%	Accuracy%	AUC^a^	Years of experience	Year of IOTA certification
A	88 (75–100)	93 (86–100)	93 (83–98)	88 (71–96)	91 (82–97)	0.907	27	2014
B	96 (88–100)	93 (86–100)	98 (86–99)	89 (73–96)	94 (86–98)	0.947	23	2016
C	92 (81–100)	91 (83–99)	95 (84–99)	85 (69–94)	91 (82–97)	0.916	14	2014
D	96 (88–100)	93 (86–100)	98 (86–99)	89 (73–96)	94 (86–98)	0.947	10	2014
E	92 (81–100)	82 (71–93)	95 (83–99)	74 (60–84)	86 (75–93)	0.871	4	2015
F	96 (88–100)	82 (71–93)	97 (84–99)	75 (61–85)	87 (77–94)	0.891	6	2016
G	72 (54–90)	80 (68–92)	84 (73–91)	67 (51–79)	77 (66–86)	0.760	5	2016
H	100 (100–100)	78 (66–90)	100 (90–100)	71 (59–81)	86 (75–93)	0.889	2	2017
I	88 (75–100)	87 (77–97)	93 (82–97)	78 (63–89)	87 (77–94)	0.873	1	2018
L	96 (88–100)	42 (28–57)	95 (73–99)	48 (41–54)	61 (49–63)	0.691	1	NA
M	96 (88–100)	44 (30–59)	95 (74–99)	49 (42–56)	63 (50–74)	0.702	1	NA

^a^Pearson correlation coefficient between AUC and year of IOTA accreditation: 0.694 (*p* = 0.018)

## Discussion

Our study evaluated interobserver agreement between examiners with different levels of experience in classifying adnexal tumors using IOTA terminology and subjective assessment, in assessing the presence of papillary projections and the quantification of Color Score.

Tumor type and the presence of papillary projections are two important aspects in the evaluation of adnexal lesions according to IOTA models. Two previous studies reported data on interobserver agreement in tumor classification and the identification of papillary projections. The first study [15], conducted by two expert observers with specialized knowledge in adnexal mass diagnosis and a deep understanding of IOTA terminology, demonstrated a high level of agreement in defining tumor types and identifying papillary projections.

Similarly, Zannoni et al. [16] reported a good agreement in tumor classification among both expert and less experienced observers, defined as trainees with at least 2 years’ training in gynecological ultrasound (Fleiss kappa 0.695 and 0.735, respectively), while agreement on the presence of papillations was moderate regardless observers’ experience, with Fleiss kappa in the range of (0.441, 0.570).

Our results differed from those reported by Zannoni et al. Specifically, we found that agreement was higher among the most expert examiners in identifying papillary projection (Fleiss kappa 0.694), compared to tumor type classification where the agreement was only moderate (Fleiss kappa 0.592). The reason of this discrepancy could lie in the histology of the lesions included in the two studies: in our study a great proportion were benign or borderline and only the 16% were invasive or metastases from tumors at other sites, while the above-mentioned paper included a greater component of invasive masses or metastasis from other tumors (33.7%), because data were collected in a referral cancer center. It could be speculated that, since invasive tumors often present a solid appearance, they may be easier to classify compared to benign and borderline tumors, producing a higher agreement among observers. Tumor type classification entails, in addition to papillary projections identification, the detection of septa, which are frequently part of benign and borderline masses and can be difficult to identify.

Observers were asked to apply IOTA Simple Rules to the tumors in analysis. Agreement with regard to classifying adnexal masses as benign, malignant or inconclusive among all observers and in group 1 and group 2 observers was moderate (Fleiss kappa 0.518 and 0.498, respectively), whereas among the four residents it was fair (Fleiss kappa 0.400). This difference in terms of agreement among the three groups was not statistically significant. Within group 3, Cohen’s kappa between the two residents with IOTA certification indicated a moderate agreement which is similar to the more experienced sonographers. Overall, observers trained to the use of IOTA models have a moderate agreement in the application of Simple Rules, regardless the years of experience in gynecological ultrasound.

Neha Antil et al. [17] recently evaluated agreement in classifying adnexal masses using Simple Rules among observers of different levels of experience (4 fellows and 4 attendings). They found that agreement calculated through interclass correlation coefficient (ICC) was excellent and, consistent with our results, it did not improve with the years of experience.

Two studies analyzed agreement in the application of Simple Rules to stored 3D volumes [18, 19] and, similarly to our study, agreement between observers was moderate: the use of 3D volumes, compared with 2D videoclips, does not seem to bring advantages in masses classification through Simple Rules.

Consistent with previous studies [20], we found that interobserver agreement in the subjective classification of tumors as benign or malignant decreased with decreasing years of experience. However, in contrast, we observed that interobserver agreement in the application of Simple Rules was not affected by years of experience*.* Faschingbauer et al. [21] tested the diagnostic performance and interobserver agreement in subjective assessment of ovarian masses using pattern recognition, in level III and level II practitioners*,* as defined in the guidelines of the European Federation of Societies for Ultrasound in Medicine and Biology [22], and trainees. Consistent with our results, they found that interobserver agreement in subjective assessment was good among the most experienced observers and it decreased with decreasing experience.

Data in literature on the agreement in Color Score assignment are controversial: according to some studies, Color Score is reproducible even in moderately experienced observers [23, 24], on the other hand Sladkevicius and Valentin reported a fair agreement in the assessment of Color Score, despite their experience in gynecological ultrasound [15]. We found that there was a fair agreement in assessing the Color Score among all observers (Cohen’s kappa 0.380), moderate among the most experienced (Cohen’s kappa 0.460) and fair in the others two subgroups (Cohen’s kappa 0.374 and 0.348). This evaluation was done in a smaller number of lesions, since Color/Power Doppler videoclips were provided for 58 out of 70 tumors. In addition, using videoclip the examiner was not able to modify Doppler settings. These two aspects of the study could have adversely affected our results.

Regarding observers accuracy assessment, our findings align with those of previous studies, which have demonstrated that expert observers can accurately differentiate between benign and malignant tumors using subjective assessment [25, 26]. In addition, our analysis of sensitivity, specificity, and area under the ROC curve showed that the accuracy of this method improves with years of experience [21]. Our analysis indicated that the most pronounced trend was observed in specificity, while sensitivity did not exhibit a significant decrease with the examiner's experience. This finding suggests that less experienced sonographers may be more likely to err on the side of caution and classify uncertain lesions as suspicious, even if they may not necessarily be malignant.

In addition, we found that observers more experienced in IOTA terminology and models were more accurate. The impact of IOTA terminology knowledge on accuracy especially emerged in group 3: the two residents with IOTA certification had comparable sensitivity and specificity to examiners with more than three years of experience in gynecological ultrasound. This finding suggests that the attendance to IOTA course with final certification could play an important role in the learning process of less experienced sonographers, improving their accuracy to distinguish between benign and malignant lesions.

The strength of this study is that interobserver agreement in two assessments developed by IOTA, Color Score assignment and evaluation of adnexal masses through Simple Rules and subjective assessment were evaluated in the same pool of eleven observers with different levels of experience. The main limitation of the present study is that the interobserver agreement was not evaluated using real-time ultrasound but by means of videoclips and that the number of observers was relatively small. However, these limitations are similar to the studies mentioned above.

We found that experience substantially impacts on accuracy and interobserver agreement in subjective assessment of ovarian lesions, while the application of Simple Rules can mitigate the role of experience on interobserver agreement in adnexal lesions evaluation. In addition, being familiar with the IOTA models seemed to improve the diagnostic accuracy among residents. Our study demonstrated that identifying papillary projections, classifying tumor types, and assigning Color Scores can lead to disagreements in identifying their respective morphological features. Specifically, we found that disagreements were less evident for papillary projections, with good agreement among examiners (measured as Fleiss kappa). However, for tumor type classification and Color Score assignment, disagreements were much more pronounced, with no more than moderate agreement achieved when measured using Fleiss and Cohen kappa.

Improvement in the assessment of adnexal masses to optimize recognition of features that are used to assess the risk of malignancy is still needed. On one hand, IOTA models and terminology have provided important guidelines to accomplish this. On the other hand, artificial intelligence (AI) and image augmentation have the potential to improve the objective assessment of features such as papillary projections and irregularity of the cyst wall, potentiating the role of IOTA models in reducing variability. These are areas for potential future research.

## Data Availability

Data are available on request from the corresponding author.
